# Place of residence and blood sugar testing practices among men: insights from the 2021 Madagascar demographic and health survey

**DOI:** 10.1186/s12889-024-19248-5

**Published:** 2024-06-25

**Authors:** Joshua Okyere, Castro Ayebeng, Barbara Sakyi, Kwamena Sekyi Dickson

**Affiliations:** 1 Department of Population and Health, University of Cape Coast, Cape Coast, Ghana; 2 School of Nursing & Midwifery, College of Health Sciences, Kwame Nkrumah University of Science and Technology, Kumasi, Ghana

**Keywords:** Diabetes, Screening, Health service research, Preventive medicine, Public Health, Madagascar

## Abstract

**Background:**

In 2021, Madagascar had approximately 13,919 people living with diabetes, with 66.1% of cases being undiagnosed. The implication is that this population are at high risk of developing diabetes complications which will affect their quality of life. However, promoting the uptake of screening practices such as the blood glucose test among the asymptomatic population would offer a chance to reduce the prevalence of undiagnosed diabetes in the country. This study examined the association between place of residence and blood sugar testing practices among men in Madagascar.

**Methods:**

Secondary data from the men recode file of the 2021 Madagascar Demographic and Health Survey (MDHS) was used. A sample of 9,035 were used for the analysis. Descriptive and multivariate analyses were performed in STATA version 14. The results are presented in adjusted odds ratio (AOR) with the corresponding 95% confidence interval.

**Results:**

Only 5.83% reported to have ever had their blood glucose/sugar tested by a health professional. Residing in rural areas was associated in lower likelihood of undergoing a test to check one’s blood sugar level [AOR = 0.23; 95%CI = 0.19–0.28] compared to those in urban areas. This association remained consistent even after adjusting for the effects of covariates [AOR = 0.67; 95%CI = 0.52–0.86].

**Conclusion:**

We conclude that place of residence plays a significant role in influencing men’s decision to test their blood glucose level. It is, therefore, imperative for the Madagascar Public Health Department to liaise with the government to bridge the rural-urban disparities in terms of accessibility to blood glucose testing services. Practically, this can be achieved by instituting community-based health services centers in the rural areas of Madagascar to mitigate the rural-urban disparities. Also, health education campaigns to raise men’s awareness about the need to test their blood glucose level must necessarily target older men, those without formal education, those without health insurance, and men who have been diagnosed with hypertension.

## Background

Non-communicable diseases (NCDs) are on the rise in all countries [1]. Type 2 diabetes mellitus is one of the increasing NCDs affecting millions of populations worldwide. Biologically, diabetes is characterised by insulin resistance and dysfunction of the β cells [2]. The 2022 report of the International Diabetes Federation indicates that there are approximately 537 million people living with diabetes worldwide; this figure is projected to increase by 46% by 2045 [3]. The report further shows that people living in low-and-middle-income countries (LMICs) constitute 4 out of 5 cases [3]. In Africa, there were nearly 24 million people living with diabetes in 2021, with 416,000 deaths attributable to the disease [3]. The International Diabetes Federation further reported that Madagascar had approximately 13,900 people living with diabetes, with 66.1% of cases being undiagnosed [3].

Late diagnosis of diabetes has been found to be associated with several complications [4, 5]. These include renal failure [6], blindness [7], cardiomyopathy and heart failure [8], stroke [9], and nonalcoholic fatty liver disease [10]. Hence, early detection and diagnosis of diabetes is essential to promote better management, survivorship and improved quality of life. One of the proven ways to facilitate early detection and diagnosis is through routine blood glucose/sugar tests [10].

The high rates of undiagnosed and its associated complications pose a significant threat to the attainment of the Sustainable Development Goals (SDGs) target 3.4 which seeks to reduce premature deaths from NCDs [11, 12]. However, promoting the uptake of screening practices such as the blood glucose/sugar test among the asymptomatic population offers a chance to reduce the prevalence of undiagnosed diabetes in the country.

In order for Madagascar’s Ministry of Public Health and other stakeholders to develop interventions that will support early detection and diagnosis of diabetes, it is imperative to know the current situation in terms of the proportion of the population that participates in routine blood glucose/sugar tests and the factors associated with their screening behaviour. Given that previous studies have identified rural residency to be associated with poor screening uptake practices for other NCDs such as prostate cancer [13, 14] and hypertension [15], we hypothesize that men residing in rural areas would be less likely to undergo blood glucose/sugar tests. Despite an extensive literature search, we were not able to identify an empirical or published study that investigated this issue. This presents an important knowledge gap that must be filled to inform policy-making in Madagascar. To provide evidence to support our hypothesis, we examined the association between place of residence and blood sugar testing practices among men in Madagascar using nationally representative data.

## Methods

### Data source and study design

For this study, we used secondary data from an earlier study, namely from the men recode file of the 2021 Madagascar Demographic and Health Survey (MDHS). In the initial sampling, the MDHS designated the administrative divisions of Madagascar, known as “regions,” as the first stratum [16]. Following this, within each region, the population was subdivided into urban and rural areas, establishing the second stratum. A total of 45 sample strata were created. The 2021 DHS sampling frame was from the 2018 Madagascar General Population and Housing Census. The MDHS utilized a two-stage sampling methodology. The sample was drawn independently from each stratum. In the first degree, 657 clusters were drawn with probability proportional to size. Each selected cluster produced a list of households [16].

Overall, 22,106 households were selected after the count, and 20,790 were identified and occupied [16]. Of these, 20,510 were interviewed at the time of the survey, resulting in a 99% household response rate. A total of 19,879 women aged 15–49 were eligible, and 18,869 were actually surveyed, yielding a response rate of 95% for eligible women, slightly higher in rural areas than in urban areas (96% vs. 93%) [16]. The men’s survey was conducted in one out of every two households: 9,930 men aged 15–59 were eligible, and 9,037 were interviewed, resulting in a response rate of 91% for eligible men. Urban areas had a slightly lower response rate for eligible men (87%) compared to rural areas (92%) [16].

### Measures

#### Dependent variable

Blood glucose/sugar test was the dependent variable. This variable was derived from the question, ‘Have you ever had blood sugar measured by a health professional?’ The original responses were ‘Yes’, ‘No’ and ‘Don’t know’. However, we dropped the ‘Don’t know’ responses (*n* = 2) from the data to have a binary outcome of ‘0 = No’ and ‘1 = Yes’.

#### Independent variable

Our independent variable was place of residence which was coded as a binary variable: ‘0’=’Urban’ and ‘1’=’Rural’.

### Covariates

A total of eight covariates were included. These were age, educational attainment, wealth index, frequency of newspaper/magazine reading, frequency of radio listening, frequency of television watching, health insurance status, and hypertension diagnosis. Age was coded as: “15–19 years”, “20–24 years”, “25–29 years”, “30–34 years”, “35–39 years”, “40–44 years”, “45–49 years”, “50–54 years”, and “55–59 years”. Education was coded to have the following responses: “No education”, “Primary”, “Secondary”, and “Higher”. The frequency of exposure to the media was categorized as “Not at all”, “Less than once a week”, and “At least once a week”. Wealth index was captured as “Poorest”, “Poorer”, “Middle”, “Richer”, and “Richest”. Health insurance was coded as “Covered” or “Not covered”, while hypertension diagnosis was coded as “Yes” or “No”.

### Data analyses

The analyses were performed in STATA version 14 (StataCorp, College Station, TX, USA). Firstly, the response ‘Don’t know’ for the outcome variable was dropped. This reduced the working data from 9,037 to 9,035. Using the weighting variable mv005, we weighted the data to offset the effects of sampling bias. Cross-tabulations and Pearson’s chi-square test (*X*^2^) were computed. A bivariable logistic regression model was fitted to examine the association between place of residence and blood glucose/sugar test uptake. After this, we fitted a multivariable logistic regression to examine the association between place of residence and blood glucose/sugar test uptake while adjusting for the effects of the covariates. Only variables with *p*-values < 0.05 were selected into the multivariable logistic regression. The findings from this model were presented in adjusted odds ratio (AOR) and the corresponding 95% confidence interval. Prior to fitting the multivariable logistic regression model, we tested for multicollinearity by checking the Variance Inflation Factor (VIF). The mean VIF result was 3.83 indicating low multicollinearity. The model with the lowest Akaike Information Criterion (AIC) was deemed the best model (i.e., Model II).

## Results

### Background characteristics of respondents

Table 1 shows the distribution of the background characteristics of the respondents. The majority were rural residents (79.58%), aged 15–19 years (21.74%), and had primary education (43.76%). Most of the respondents did not read newspapers or magazines at all (83.40%), listened to radio at least once a week (40.73%), and did not watch television at all (67.00%). The majority were in the richer wealth index (22.63%). The majority lacked health insurance cover (96.52%). Only 7.30% had been diagnosed with hypertension.

### Proportion of men who test for blood sugar

Our findings reveal low uptake of blood sugar tests among men; only 5.8% reported to have ever undergone this test (see Table 1). The proportion of blood sugar tests was relatively high among men who resided in urban areas (13.78%), those aged 55–59 years (12.98%), men with higher educational attainment (27.79%), those in the richest wealth index (15.09%), those who had health insurance coverage (25.91%), and those who had ever been diagnosed with hypertension (18.51%). Also, higher testing was observed among men who read newspapers/magazines (17.60%), listened to the radio (8.64%) or watched TV (16.53%) at least once a week (see Table 1).

**Table 1:** Distribution of blood sugar test uptake across the main explanatory variable and covariates

		Blood sugar tested	
Variables	Weighted sample (*n*) I think you need to add %	Proportion that tested their blood sugar *n* (%)	Chi-square (X^2^); *p*-value
**Place of residence**			*X* ^**2**^ ** = 292.0503; ** ***p*** ** < 0.001**
Urban	1845 (20.42)	254 (13.78)	
Rural	7190 (79.58)	273 (3.79)	
**Age**			***X***^**2**^ **= 183.2624; *p* < 0.001**
15–19 years	1965 (21.74)	32 (1.64)	
20–24 years	1463 (16.19)	43 (2.96)	
25–29 years	1239 (13.72)	78 (6.29)	
30–34 years	987 (10.92)	56 (5.63)	
35–39 years	898 (9.94)	59 (6.55)	
40–44 years	787 (8.71)	68 (8.67)	
45–49 years	686 (7.59)	69 (9.96)	
50–54 years	562 (6.23)	64 (11.45)	
55–59 years	448 (4.96)	58 (12.98)	
**Educational level**			***X***^**2**^ **= 506.0919; *p* < 0.001**
No education	1270 (14.06)	18 (1.44)	
Primary	3954 (43.76)	128 (3.23)	
Secondary	3386 (37.48)	263 (7.77)	
Higher	425 (4.70)	118 (27.79)	
**Frequency of reading newspaper/magazine**			***X***^**2**^ **= 196.8802; *p* < 0.001**
Not at all	7536 (83.40)	328 (4.35)	
Less than once a week	1125 (12.46)	133 (11.83)	
At least once a week	374 (4.14)	66 (17.60)	
**Frequency of listening to radio**			***X***^**2**^ **= 139.8637; *p* < 0.001**
Not at all	3048 (33.74)	85 (2.79)	
Less than once a week	2307 (25.54)	124 (5.37)	
At least once a week	3680 (40.73)	318 (8.64)	
**Frequency of watching television**			***X***^**2**^ **= 480.8646; *p* < 0.001**
Not at all	6053 (67.00)	169 (2.80)	
Less than once a week	1325 (14.66)	84 (6.32)	
At least once a week	1657 (18.34)	274 (16.53)	
**Wealth index**			***X***^**2**^ **= 433.2572; *p* < 0.001**
Poorest	1439 (15.93)	13 (0.91)	
Poorer	1712 (18.95)	27 (1.56)	
Middle	1819 (20.14)	70 (3.84)	
Richer	2045 (22.63)	113 (5.51)	
Richest	2020 (22.35)	305 (15.09)	
**Insurance status**			***X***^**2**^ **= 261.8573; *p* < 0.001**
Not covered	8721 (96.52)	446 (5.11)	
Covered	314 (3.48)	81 (25.91)	
**Ever diagnosed with hypertension**			***X***^**2**^ **= 228.4065; *p* < 0.001**
No	8375 (92.70)	405 (4.83)	
Yes	660 (7.30)	122 (18.51)	
**Total**	**9035**	**527 (5.83%)**	

### Association between place of residence and blood sugar testing

Residing in rural areas was associated with lower likelihood of undergoing a test to check one’s blood sugar level [AOR = 0.23; 95%CI = 0.19–0.28] compared to those in urban areas. This association remained consistent after adjusting for the effects of the covariates [AOR = 0.67; 95%CI = 0.52–0.86]. Men of younger age were less likely to get their blood glucose tested than those aged 55–59 years. Other covariates associated with higher odds of testing blood glucose included having secondary [AOR = 2.39; 95%CI = 1.44–3.98] or higher education [AOR = 5.52; 95%CI = 3.14–9.71], being in the richest wealth index [AOR = 2.19; 95%CI = 1.22–3.96], having health insurance coverage [AOR = 1.74; 95%CI = 1.26–2.39], and having been diagnosed with hypertension [AOR = 2.08; 95%CI = 1.60–2.71]. Listening to the radio [AOR = 1.34; 95CI = 1.01–1.79] and watching TV [AOR = 2.40; 95%CI = 1.74–3.31] at least once a week were associated with higher likelihood of checking one’s blood sugar (see Table 2).

**Table 2:** Association between place of residence and blood sugar testing

Variables	Model I OR	Model II AOR
**Place of residence**		
Urban	Ref.	Ref.
Rural	0.23 [0.19–0.28]***	0.67 [0.52–0.86]**
**Age**		
15–19 years		0.11 [0.07–0.18]***
20–24 years		0.21 [0.13–0.32]***
25–29 years		0.38 [0.26–0.57]***
30–34 years		0.45 [0.29–0.68]***
35–39 years		0.41 [0.27–0.63]***
40–44 years		0.63 [0.41–0.95]*
45–49 years		0.74 [0.49–1.11]
50–54 years		0.87 [0.57–1.31]
55–59 years		Ref.
**Educational level**		
No education		Ref.
Primary		1.54 [0.94–2.53]
Secondary		2.39 [1.44–3.98]***
Higher		5.52 [3.14–9.71]***
**Frequency of reading newspaper/magazine**		
Not at all		Ref.
Less than once a week		1.18 [0.92–1.53]
At least once a week		1.03 [0.73–1.45]
**Frequency of listening to radio**		
Not at all		Ref.
Less than once a week		1.30 [0.95–1.79]
At least once a week		1.34 [1.01–1.79]*
**Frequency of watching television**		
Not at all		Ref.
Less than once a week		1.45 [1.06–1.99]*
At least once a week		2.40 [1.74–3.31]***
**Wealth index**		
Poorest		Ref.
Poorer		1.29 [0.72–2.31]
Middle		2.16 [1.26–3.70]**
Richer		2.32 [1.36–3.98]**
Richest		2.19 [1.22–3.96]**
**Insurance status**		
Not covered		Ref.
Covered		1.74 [1.26–2.39]***
**Ever diagnosed with high blood pressure**		
No		Ref.
Yes		2.08 [1.60–2.71]***
***Model Fitness***		
**Pseudo R** ^**2**^	0.0626	0.2052
**Prob > chi2**	< 0.001	< 0.001
**AIC**	3728.521	3207.687

^*^p ≤ 0.05, **p ≤ 0.01, ^***^p ≤ 0.001; OR: Odds ratio; AOR: Adjusted odds ratio

## Discussion

This study was conducted using nationally representative data to investigate the role of place of residence in the practice of blood sugar testing among men aged 15–59 years in Madagascar. To the best of our knowledge, this is the first multivariate logistic regression model conducted to disentangle the complex associations between residential location and the practice of blood sugar testing, adjusting for other relevant covariates among men aged 15–59 years in Madagascar.

Our analysis shows that, out of the 9,035 men involved in the study, only 5.8% had their blood sugar levels tested. This low screening uptake has the propensity to increase the prevalence of undiagnosed diabetes in Madagascar, which may adversely impact the country’s efforts and progress to achieving the SGD target 3.4.

Evidence from the results supports our hypothesis that there is a significant association between place of residence and blood glucose/sugar testing practices. Thus, those residing in rural areas were significantly less likely to have their blood sugar level tested. Often, the available supplies and equipment to render the service may be concentrated in hospitals in urban areas, which may not be convenient to those residing in rural settings. For instance, a study conducted in Tanzania yielded similar results, suggesting that most services for NCDs were primarily available at the hospital level [17], which are mainly located in urban settings. Also, men residing in rural areas may have lower levels of awareness about the importance of regular health check-ups, including blood sugar screening, compared to urban populations, which could stem from limited access to health information.

Among the covariates, increasing age was found to be a facilitating factor for men’s uptake of blood glucose/sugar testing. Compared to men aged 55–59 years, those of younger age were significantly less likely to get their blood glucose/sugar level tested. This result is expected as studies have established the positive relationship between older age and the risk of type 2 diabetes [18–20]. Therefore, it is possible that men of younger age may perceive themselves as at a lower risk of developing the disease. As opined by the health belief model [21], high perceived susceptibility serves as push factor for individuals to take up health preventive measures.

It was observed that as the educational attainment of the respondents increased, the probability of utilizing blood sugar tests also increased. This finding is analogous to a previous study [22] that found a significant association between higher educational attainment and higher diabetes screening practice. One possible explanation for this may be that men with some level of formal education are more likely to be exposed to health information about the relevance of screening modalities like blood glucose/sugar testing in promoting the early detection and treatment of diabetes.

Exposure to the media, such as radio and television, at least once a week increased the likelihood of individuals testing their blood sugar levels. As postulated by the health belief model [21], individuals would take up health preventive measures when they perceive that the benefits outweigh the cost. Through frequent media exposure, men are informed of the benefits of getting their blood glucose/sugar levels tested. This is likely to make them more health conscious, and dispelling misperceptions that they might hold about testing one’s blood glucose/sugar level. Hence, it is imperative for the Madagascar Ministry of Public Health to leverage the opportunities that TV and radio presents in terms of health information dissemination and awareness creation about the need to participate in blood glucose/sugar testing.

Respondents who were covered by health insurance had significantly higher odds of testing their blood sugar levels. Despite the commendable progress achieved by several SSA countries like Madagascar in their pursuit of universal healthcare access and coverage, financial constraints still hinder the utilisation of diabetes services. For instance, prior studies conducted in Ghana [23] and Tanzania [17] revealed that individuals often forgo blood sugar tests due to financial constraints, as this test is typically not covered under most standard medical insurance policies. Therefore, the findings that health insurance coverage increases the likelihood of undergoing blood glucose/sugar testing underscores a need for Madagascar to expand health insurance coverage.

The analysis revealed that individuals who had ever been diagnosed with hypertension were significantly more likely to have their blood sugar levels tested. This observation is substantiated by the well-established association between hypertension and diabetes, which are two common chronic conditions that often coexist [24, 25]. Insulin resistance can elevate blood sugar levels, potentially damaging blood vessels and contributing to hypertension [26]. Given the heightened diabetes risk among those with high blood pressure, healthcare providers are more likely to recommend diabetes screening for individuals with hypertension compared to those who are normotensive.

### Implications for policy and practices

Our study underscores a need for policymakers to consider expanding the availability of blood sugar screening supplies and equipment to rural healthcare facilities, coupled with efforts to educate rural populations about the importance of regular health check-ups. Additionally, public health campaigns should focus on younger age groups, as the study reveals that older men are more likely to undergo testing. Educational programs should also be intensified, targeting individuals with lower levels of formal education to bridge the knowledge gap. Furthermore, the role of mass media in promoting testing behaviour should not be underestimated, and efforts should be made to enhance media campaigns promoting diabetes screening. Lastly, expanding health insurance coverage for blood sugar tests is essential to reduce financial barriers, as demonstrated by the higher testing rates among those with insurance.

### Strengths and limitations

This study was based on a nationally representative data, which enhances the generalizability of the findings to the studied population in Madagascar. Moreover, the present study is arguably the first study of its kind in Madagascar that examined how place of residence influences blood glucose/sugar testing among men thus expanding the frontiers of what is already known about screening for diabetes. Notwithstanding, the study was limited by the cross-sectional nature of the MDHS which precludes us from establishing causal relationships. Secondly, the data are self-reported and thus introduces the possibility of recall bias or social desirability bias. Also, the study primarily focuses on men aged 15–59 years, potentially missing valuable insights into blood sugar testing behavior among women and older men (60+). Our reliance on a secondary dataset was also a significant limitation as we were limited to only variables within the dataset. Consequently, other key confounders could not be accounted for in our analysis. These include the perceived benefits, perceived barriers, level of perceived susceptibility, level of engagement in physical activity, and obesity status.

## Conclusion

We conclude that place of residence plays a significant role in influencing men’s decision to test their blood glucose level. It is, therefore, imperative for the Madagascar Public Health Department to liaise with the government to bridge the rural-urban disparities in terms of accessibility to blood glucose testing services. Practically, this can be achieved by instituting community-based health services centers in the rural areas of Madagascar to mitigate the rural-urban disparities. Also, health education campaigns to raise men’s awareness about need to test their blood glucose level must necessarily target older men, those without formal education, those without health insurance, and men who have been diagnosed with hypertension.

## Data Availability

The Madagascar Demographic and Health Survey is freely available at: http://www.dhsprogram.com/data/available-datasets.cfm.

## Abbreviations

AOR: Adjusted Odds Ratio
IDF: International Diabetes Federation
LMICs: Low-and-middle-income Countries
NCDs: Non-communicable Diseases
OR: Odds Ratio
VIF: Variance Inflation Factor
